# Associação entre a Função Renal e a Incidência de Desfechos Cardiovasculares Maiores em 1 Ano Após o Primeiro Infarto Agudo do Miocárdio

**DOI:** 10.36660/abc.20250082

**Published:** 2025-07-29

**Authors:** Daniel Medeiros Moreira, Marco Antônio de Sousa, Maria Fernanda Scarduelli Cechinel, Roberto Léo da Silva, Tammuz Fattah, Rodrigo de Moura Joaquim

**Affiliations:** 1 Universidade do Sul de Santa Catarina Palhoça SC Brasil Universidade do Sul de Santa Catarina, Palhoça, SC – Brasil; 2 Instituto de Cardiologia de Santa Catarina São José SC Brasil Instituto de Cardiologia de Santa Catarina, São José, SC – Brasil

**Keywords:** Rim, Creatinina, Infarto do Miocárdio

## Abstract

**Fundamento:**

As doenças cardiovasculares constituem a principal causa de mortalidade no mundo, gerando impactos sociais e econômicos significativos. Essas condições frequentemente coexistem com comorbidades, como a disfunção renal.

**Objetivos:**

Avaliar a associação entre a depuração de creatinina e a incidência de desfechos cardiovasculares no período de 1 ano em pacientes com diagnóstico de infarto agudo do miocárdio (IAM).

**Métodos:**

Este estudo de coorte prospectivo incluiu pacientes hospitalizados em decorrência do primeiro episódio de IAM. A depuração de creatinina foi analisada em relação aos desfechos cardiovasculares, incluindo recidiva de IAM, acidente vascular cerebral e óbito de causa cardiovascular. Valores de p <0,05 foram considerados estatisticamente significativos.

**Resultados:**

Foram analisados 1.324 pacientes, com idade média de 60,9 ± 11,4 anos, dos quais 67,4% do sexo masculino. Depuração de creatinina inferior a 60 ml/min apresentou associação significativa com hipertensão arterial sistêmica (79,6% vs. 55,1%; p<0,001), diabetes mellitus (40,8% vs. 24,5%; p<0,001) e dislipidemia (38,8% vs. 31,4%; p=0,043). Valores mais elevados de depuração de creatinina associaram-se a menor risco de eventos adversos cardiovasculares maiores (MACEs) em 1 ano (HR: 0,992; IC 95%: 0,984-0,999; p=0,030). Ademais, verificou-se associação com menor mortalidade geral (HR: 0,984; IC 95%: 0,970-0,998; p=0,021).

**Conclusão:**

Valores mais elevados de depuração de creatinina associaram-se a menores razões de risco para MACEs e mortalidade geral no período de 1 ano após o IAM.

## Graphical abstract

**Figura Central:**
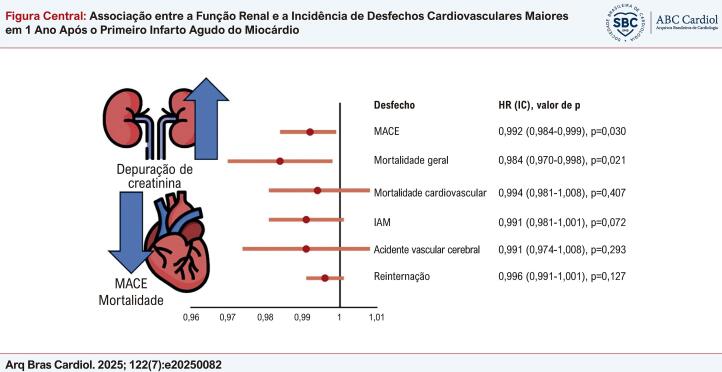
Associação entre a Função Renal e a Incidência de Desfechos Cardiovasculares Maiores em 1 Ano Após o Primeiro Infarto Agudo do Miocárdio

## Introdução

A doença renal crônica (DRC) é definida por alterações na função e/ou estrutura renal, caracterizando-se por sua irreversibilidade e progressão gradual.^1^ A DRC é uma condição de alta prevalência, afetando mais de 800 milhões de pessoas em todo o mundo, frequentemente associada a outras enfermidades, incluindo as doenças cardiovasculares (DCV).^2,3^ O diagnóstico baseia-se, predominantemente, em exames laboratoriais e em estimativas como a taxa de filtração glomerular (TFG), calculada a partir de marcadores de filtração, como a creatinina sérica, ajustada por idade, sexo e etnia.^4,5^

As DCV constituem a principal causa de morbimortalidade em indivíduos com disfunção renal, englobando condições como a doença arterial coronariana (DAC), aterosclerose, angina pectoris, infarto agudo do miocárdio (IAM), acidente vascular cerebral (AVC) e morte súbita cardíaca. No Brasil, as DCV configuram-se como a principal causa de óbito, destacando-se o AVC e a DAC como os principais contribuintes — frequentemente relacionados a fatores de risco modificáveis, como tabagismo, obesidade, alimentação inadequada e sedentarismo.^6-8^

A doença renal associa-se de forma independente ao aumento do risco cardiovascular, sendo a TFG e a albuminúria indicadores-chave dessa relação.^9,10^ Ademais, a DRC relaciona-se tanto a causas ateroscleróticas quanto não ateroscleróticas de DCV.^11^ Fatores de risco cardiovascular tradicionais, como diabetes mellitus (DM) e hipertensão arterial sistêmica (HAS), também contribuem para o desenvolvimento de doença renal.^12^ Eventos adversos cardiovasculares maiores (MACEs), como o IAM, são mais frequentes em indivíduos com disfunção renal.^13^ Além disso, há um reconhecimento crescente do papel de fatores de risco não tradicionais — como os níveis de ácido úrico e fósforo, que podem ser influenciados pela DRC — na fisiopatologia das DCV.^14^

O presente estudo tem como objetivo investigar a relação entre a função renal e a incidência de MACEs em pacientes após IAM ao longo de 1 ano de seguimento.

## Métodos

Este estudo constitui uma subanálise da coorte prospectiva *Catarina Heart Study*, que incluiu pacientes com primeiro episódio de IAM, conforme definido pela *Third Universal Definition of Myocardial Infarction* — vigente à época da concepção da coorte.^15^ Foram incluídos indivíduos de ambos os sexos, com idade ≥18 anos, admitidos com suspeita de IAM, caracterizada por dor precordial e elevação do segmento ST no ponto J em duas derivações contíguas (≥0,1 mV na maioria das derivações; para V2–V3: ≥0,2 mV em homens ≥40 anos, ≥0,25 mV em homens <40 anos e ≥0,15 mV em mulheres). Outro critério de inclusão foi a presença de dor precordial associada à elevação da troponina I acima do percentil 99 do limite superior de referência. Embora a creatinoquinase MB tenha sido considerada inicialmente para confirmação diagnóstica, ela não foi utilizada na definição de IAM em nenhum dos casos incluídos na presente análise. Pacientes com histórico prévio de IAM foram excluídos do estudo.^16^

O estudo avaliou a relação entre a depuração de creatinina e diversos desfechos clínicos. O desfecho primário foi a ocorrência de MACEs, definido como morte cardiovascular, novo AMI ou AVC. Os desfechos secundários incluíram cada um desses componentes individualmente, além de angina instável, reinternação hospitalar em até 1 ano, trombose aguda de *stent* e reestenose de *stent*. Também foi avaliada a associação entre disfunção renal (TFG <60 ml/min) e fatores de risco, como HAS, DM, dislipidemia, sedentarismo, tabagismo e história familiar de DCV. Os dados foram coletados por meio de formulário padronizado desenvolvido para o *Catarina Heart Study*, a ser preenchido pelo médico assistente. Este formulário contempla dados sociodemográficos, medidas antropométricas, hábitos alimentares, informações clínicas, avaliação do estado mental, procedimentos realizados nas primeiras 24 horas de internação, índice de religiosidade, nível de atividade física, status hemodinâmico, resultados de ecocardiograma, exames laboratoriais e informações de seguimento em 1 ano.

A depuração de creatinina foi calculada utilizando a fórmula de Cockcroft,^17^ com base na primeira dosagem de creatinina obtida nas primeiras 72 horas após a admissão hospitalar. Os valores contínuos de depuração de creatinina foram utilizados para a análise dos desfechos primário e secundários. Para as comparações entre função renal e fatores de risco cardiovascular, os pacientes foram categorizados em dois grupos: aqueles com disfunção renal (TFG <60 ml/min) e aqueles com função renal preservada (TFG ≥60 ml/min).^18^

O cálculo do tamanho amostral indicou a necessidade de incluir 458 pacientes, assegurando um poder estatístico de 90% e nível de significância de 5%, para detectar uma incidência de 24% de MACEs em pacientes com disfunção renal, em comparação a 12% naqueles sem disfunção, conforme dados relatados por Gallacher et al.^19^

O estudo foi aprovado por comitê de ética institucional e seguiu todas as normas éticas e legais vigentes.

### Análise estatística

Os dados foram inseridos em planilhas do Microsoft Excel e posteriormente analisados utilizando o software SPSS, versão 13.0 (Chicago: SPSS Inc.; 2005). Foram realizadas estatísticas descritivas para todas as variáveis. Os dados categóricos foram apresentados em frequências absolutas e relativas, enquanto os dados contínuos foram expressos por medidas de tendência central (média ou mediana) e suas respectivas medidas de dispersão (desvio padrão ou intervalo interquartílico [IIQ]). O teste de Kolmogorov-Smirnov foi utilizado para verificar a normalidade das variáveis contínuas. Na análise bivariada, as associações entre variáveis dependentes e independentes foram avaliadas por meio do teste do qui-quadrado. A associação entre a função renal e os desfechos clínicos foi analisada utilizando regressão de Cox, incorporando a depuração de creatinina como variável contínua (calculada pela fórmula de Cockcroft-Gault). O modelo também incluiu as variáveis DM, dislipidemia, HAS, histórico familiar de DCV, tabagismo, sedentarismo, índice de massa corporal (IMC) e idade. Considerou-se valor de p <0,05 como estatisticamente significativo.

## Resultados

Foram incluídos no estudo 1.324 pacientes que apresentaram o primeiro episódio de IAM entre 2016 e 2023. A média de idade foi de 60,9 ± 11,4 anos, e 67,4% dos participantes eram do sexo masculino. Na coorte, 58,9% apresentavam HAS, 27,1% tinham DM, 32,7% dislipidemia, 32,3% eram tabagistas e 45,9% possuíam histórico familiar de DAC. A mediana da depuração de creatinina foi de 89,5 ml/min (IIQ: 66,2-116,6). As características adicionais dos pacientes estão descritas na Tabela 1.

**Tabela 1:** – Características basais dos pacientes

Características	N (%)
FEVE, média±DP	51,3±12,5
Idade (anos), média±DP	60,9±11,4
Consumo de álcool	418 (31,6)
Diabetes melito	358 (27,1)
Dislipidemia	431 (32,7)
Hipertensão arterial sistêmica	778 (58,9)
História familiar	606 (45,9)
Sedentarismo	818 (61,9)
Sexo masculino	893 (67,4)
Tabagismo	421 (32,3)
Depuração de creatinina (ml/min), mediana (IIQ)	89,5 (66,2-116,6)
FEVE, mediana (IIQ)	53,00 (43,0-61,0)
IMC (kg/m^2^), mediana (IIQ)	27,3 (24,5-30,5)
Escore SYNTAX, mediana (IIQ)	13,00 (7,0-20,0)

DAC: doença arterial coronariana; DP: desvio padrão; FEVE: fração de ejeção do ventrículo esquerdo; IMC: índice de massa corporal; IIQ: intervalo interquartílico.

A depuração de creatinina inferior a 60 ml/min esteve associada a maior prevalência de HAS e DM. Informações detalhadas estão apresentadas na Tabela 2.

**Tabela 2:** – Associação entre depuração de creatinina e fatores de risco

Fatores de risco	Depuração <60 ml/min, n (%)	Depuração >60 ml/min, n (%)	Valor de p
Consumo de álcool	38 (19,5)	373 (34,0)	<0,001
Diabetes melito	80 (40,8)	268 (24,5)	<0,001
Dislipidemia	76 (38,8)	344 (31,4)	0,043
Hipertensão arterial sistêmica	156 (79,6)	604 (55,1)	<0,001
História familiar	70 (35,7)	523 (47,7)	0,002
Tabagismo	45 (23,2)	364 (33,6)	0,004
Sedentarismo	34 (28,3)	455 (38,7)	0,025
Sexo masculino	143 (73,0)	732 (66,5)	0,077

Na análise dos desfechos em 1 ano, observou-se que valores mais elevados de depuração de creatinina associaram-se a uma razão de risco (HR) significativamente menor para MACEs (HR: 0,992 a cada aumento de 1 ml/min; IC 95%: 0,984-0,999; p=0,030). Da mesma forma, maiores valores de depuração de creatinina estiveram associados a menor HR para mortalidade por todas as causas. Outras associações estão detalhadas na Tabela 3 e na Figura Central.

**Tabela 3:** – Associação entre valores de depuração e desfechos em 1 ano

Desfechos	Razão de risco (IC 95%)	Valor de p
Angina instável	1,002 (0,993-1,011)	0,204
Acidente vascular cerebral	0,991 (0,974-1,008)	0,293
IAM	0,991 (0,981-1,001)	0,072
MACE	0,992 (0,984-0,999)	0,030
Mortalidade cardiovascular	0,994 (0,981-1,008)	0,407
Mortalidade por todas as causas	0,984 (0,970-0,998)	0,021
Reestenose de stent	0,996 (0,979-1,014)	0,617
Reinternação	0,996 (0,991-1,001)	0,127
Trombose aguda de stent	1,002 (0,990-1,014)	0,763

IAM: infarto agudo do miocárdio; IC: intervalo de confiança; MACE: eventos adversos cardiovasculares maiores.

## Discussão

O presente estudo apresenta achados relevantes sobre a incidência de mortalidade e desfechos cardiovasculares em 1 ano em pacientes brasileiros com diagnóstico de IAM, estratificados de acordo com a depuração de creatinina na admissão. Os resultados demonstraram uma associação significativa entre a função renal comprometida e o aumento da HR tanto para mortalidade por todas as causas quanto para MACEs.

A amostra de pacientes foi predominantemente composta por indivíduos do sexo masculino, com idade média em torno de 61 anos, e mais da metade apresentava HAS — achados consistentes com estudos prévios.^20-23^ Em relação aos valores de depuração de creatinina, estudos anteriores que avaliaram a função renal em pacientes após IAM relataram medianas inferiores às observadas no presente estudo, variando entre 63,7 e 77 ml/min/1,73 m^2^. ^22,24-26^ A associação entre DM, HAS e disfunção renal observada neste estudo era esperada. O DM constitui um dos principais fatores etiológicos de lesão renal, achado que corrobora relatos prévios.^27,28^ A HAS pode atuar tanto como causa quanto como consequência do declínio da função renal, sendo ambas condições reconhecidamente fatores de risco independentes para DCV.^29^ A associação entre dislipidemia e doença renal pode ser explicada por um possível vínculo com o aumento da aterosclerose, incluindo a aterosclerose renal. No entanto, também é possível que tal associação seja espúria e decorrente do acaso.^30,31^

Esta é uma das maiores investigações brasileiras a demonstrar que a função renal na admissão representa um fator prognóstico em pacientes com IAM. Valores mais elevados de depuração de creatinina associaram-se a menores HRs para MACEs e mortalidade por todas as causas. Essa associação foi confirmada por meio de análise multivariada, a qual incluiu variáveis como DM, idade e IMC — potenciais fatores de confusão — e considerou os valores absolutos de depuração de creatinina. Em outras palavras, a menor depuração de creatinina em pacientes com IAM configura-se como preditor independente de piores desfechos, independentemente da presença de disfunção renal manifesta.

Pesquisas prévias já indicam que a função renal na admissão está associada a pior prognóstico em pacientes com IAM. Evidências demonstram que a concentração de creatinina sérica na admissão constitui um forte preditor de mortalidade intra-hospitalar em pacientes com IAM com supradesnivelamento do segmento ST, especialmente entre indivíduos mais jovens e mulheres.^32^ Outro estudo evidenciou que uma TGE estimada (TFGe) inferior a 60 ml/min/1,73 m^2^ na admissão associa-se ao aumento da mortalidade precoce e tardia em pacientes com IAM que desenvolvem lesão renal aguda (LRA).^33^ Adicionalmente, a depuração de creatinina calculada na admissão foi identificada como preditor independente de mortalidade a longo prazo em pacientes com IAM, com aumento do risco de óbito em até 10 anos após o evento.^34^ A redução da função renal na admissão também tem sido associada a maiores taxas de mortalidade em pacientes submetidos a intervenção coronária percutânea.^35^

Por outro lado, alguns estudos, embora tenham demonstrado uma associação entre menores valores de TFG e maior mortalidade em 30 dias após o IAM, também observaram que as variações percentuais na depuração não apresentaram associação significativa.^26^ Além disso, outros autores exploraram relações mais indiretas entre a disfunção renal e MACE, as quais podem ser explicadas pela presença de comorbidades frequentemente encontradas em pacientes com LRA, como a anemia, que também contribuem para piores desfechos.^36^

Esses dados reforçam a relevância da avaliação da função renal, frequentemente negligenciada no manejo de pacientes com IAM. A associação entre a função renal na admissão e os desfechos clínicos sugere que a avaliação da depuração de creatinina pode atuar como ferramenta prognóstica precoce, possibilitando a identificação de pacientes com maior risco para MACEs e mortalidade, e permitindo, assim, intervenções mais direcionadas que possam melhorar os desfechos a longo prazo.

Diversas limitações do presente estudo devem ser reconhecidas. Primeiramente, trata-se de uma análise exploratória de um banco de dados existente, o que pode implicar limitações inerentes quanto à completude e consistência dos dados, uma vez que o banco de dados não foi originalmente concebido para responder às questões de pesquisa deste estudo. Apesar de os pesquisadores terem recebido orientação e treinamento adequados, a coleta de dados pode ter sido suscetível a vieses de mensuração, em virtude das dificuldades inerentes à realização de entrevistas em ambiente de emergência.

Embora o cálculo amostral tenha indicado uma amostra suficientemente ampla, não foi realizada uma análise formal de poder estatístico, o que pode ter limitado a capacidade do estudo em detectar efeitos de menor magnitude em alguns desfechos secundários. Além disso, pacientes em estado crítico não foram incluídos, devido à impossibilidade de participação no processo de entrevista, o que pode ter introduzido viés de seleção e limitado a generalização dos resultados, especialmente em relação aos pacientes com IAM em condições mais graves.

Além disso, determinados biomarcadores associados ao risco cardiovascular, como albuminúria, níveis de ácido úrico e fósforo, não estavam disponíveis no banco de dados, o que limitou a possibilidade de avaliar sua contribuição para o prognóstico pós IAM. Também podem ter ocorrido fatores de confusão não mensurados, como adesão ao tratamento medicamentoso ou condição socioeconômica, que potencialmente influenciaram as associações observadas.

Por fim, não se pode descartar a possibilidade de erro tipo 1, uma vez que os resultados podem ter sido influenciados pelo acaso ou por vieses desconhecidos. Ademais, pode ter ocorrido confundimento por indicação, dado que pacientes com pior função renal possivelmente receberam estratégias terapêuticas distintas, as quais podem ter impactado os desfechos de forma independente.

## Conclusão

Os achados deste estudo indicam que valores mais elevados de depuração de creatinina na admissão estão diretamente associados a menor risco de MACEs e mortalidade por todas as causas no período de 1 ano pós IAM. Esses resultados sugerem que a função renal, avaliada especificamente por meio da depuração de creatinina, pode constituir um marcador prognóstico relevante para a predição de desfechos cardiovasculares a longo prazo em pacientes com IAM. Dada a simplicidade e o baixo custo da medição da depuração de creatinina, sua integração à prática clínica de rotina poderia auxiliar na identificação precoce de pacientes de alto risco que possam se beneficiar de monitoramento e intervenções terapêuticas mais intensivas.
