# Supplementary material for: Associação entre a Função Renal e a Incidência de Desfechos Cardiovasculares Maiores em 1 Ano Após o Primeiro Infarto Agudo do Miocárdio
Source: Arq Bras Cardiol. 2025 Jul 29;122(7):e20250082. [Article in Portuguese] doi: 10.36660/abc.20250082 (PMC12695091; doi:10.36660/abc.20250082)
Supplement: Translation

# Translation: Association between Renal Function and the Incidence of Major Adverse Cardiovascular Outcomes 1 Year After the First Acute Myocardial Infarction

**DOI:** 10.36660/abc.20250082i

**Authors:** Daniel Medeiros Moreira, Marco Antônio de Sousa, Maria Fernanda Scarduelli Cechinel, Roberto Léo da Silva, Tammuz Fattah, Rodrigo de Moura Joaquim

**Affiliations:** 1 Brazil Universidade do Sul de Santa Catarina, Palhoça, SC – Brazil; 2 Brazil Instituto de Cardiologia de Santa Catarina, São José, SC – Brazil

**Keywords:** Kidney, Creatinine, Myocardial Infarction

## Abstract

**Background:**

Cardiovascular diseases are the leading cause of death worldwide, with substantial social and economic impacts. These conditions are frequently associated with comorbidities, including renal dysfunction.

**Objectives:**

To evaluate the association between creatinine clearance and the incidence of cardiovascular outcomes within 1 year in patients diagnosed with acute myocardial infarction (AMI).

**Methods:**

This prospective cohort study included patients hospitalized for their first AMI. Creatinine clearance was assessed in relation to cardiovascular outcomes, including recurrent AMI, stroke, and cardiovascular death. A p-value of <0.05 was considered statistically significant.

**Results:**

A total of 1,324 patients were analyzed, with a mean age of 60.9 ± 11.4 years; 67.4% were male. Creatinine clearance <60 mL/min was significantly associated with systemic arterial hypertension (79.6% vs. 55.1%, p<0.001), diabetes mellitus (40.8% vs. 24.5%, p<0.001), and dyslipidemia (38.8% vs. 31.4%, p=0.043). Higher creatinine clearance values were associated with a reduced risk of major adverse cardiovascular events (MACE) at 1 year (HR: 0.992; 95% CI: 0.984-0.999; p=0.030). Additionally, higher clearance was linked to lower overall mortality (HR: 0.984; 95% CI: 0.970-0.998; p=0.021).

**Conclusion:**

Higher creatinine clearance values are associated with a lower hazard ratio for MACE and overall mortality within 1 year following AMI.

## Introduction

Chronic kidney disease (CKD) is defined by alterations in kidney function and/or structure, marked by its irreversibility and gradual progression.^1^ CKD is a highly prevalent condition, affecting more than 800 million people worldwide, and is frequently associated with other health conditions, including cardiovascular disease (CVD).^2,3^ Diagnosis typically involves laboratory tests and estimations such as the glomerular filtration rate (GFR), which is calculated based on filtration markers like serum creatinine, adjusted for age, sex, and ethnicity.^4,5^

CVD is a leading cause of morbidity and mortality in individuals with renal dysfunction, encompassing conditions such as coronary artery disease (CAD), atherosclerosis, angina pectoris, acute myocardial infarction (AMI), stroke, and sudden cardiac death. In Brazil, CVD is the primary cause of death, with stroke and CAD as the main contributors — often linked to modifiable risk factors such as smoking, obesity, poor diet, and a sedentary lifestyle.^6-8^

Renal disease is independently associated with increased cardiovascular risk, with GFR and albuminuria serving as key indicators of this relationship.^9,10^ Furthermore, CKD is linked to both atherosclerotic and non-atherosclerotic causes of CVD.^11^ Common cardiovascular risk factors, such as diabetes mellitus (DM) and systemic arterial hypertension (SAH), also play a role in the development of renal disease.^12^ Major adverse cardiovascular events (MACEs), such as AMI, are more frequent in individuals with renal dysfunction.^13^ Additionally, there is growing recognition of the role of nontraditional risk factors — such as uric acid and phosphate levels, which may be influenced by CKD — in the pathophysiology of CVD.^14^

The current study aims to investigate the relationship between renal function and the incidence of MACEs in patients after an AMI over the course of 1 year.

## Methods

This study is a subanalysis of the prospective cohort Catarina Heart Study, which enrolled patients with their first AMI as defined by the Third Universal Definition of Myocardial Infarction — the standard in place when the cohort was designed.^15^ Eligible participants were men and women aged ≥18 years admitted with suspected AMI characterized by precordial pain and new ST-segment elevation at the J point in two contiguous leads (≥0.1 mV in most leads; for V2–V3: ≥0.2 mV in men ≥40 years, ≥0.25 mV in men <40 years, and ≥0.15 mV in women). An additional inclusion criterion was precordial pain plus troponin I elevation above the 99th percentile of the upper reference limit. Although creatine kinase MB was originally considered for diagnostic confirmation, it was not used to define AMI in any case included in this analysis. Patients with a history of prior AMI were excluded.^16^

The study assessed the relationship between creatinine clearance and various clinical outcomes. The primary outcome was the occurrence of a MACE, defined as cardiovascular death, new AMI, or stroke. Secondary outcomes included each of these components individually, as well as unstable angina, hospital readmission within 1 year, acute stent thrombosis, and stent restenosis. The relationship between renal dysfunction (GFR <60 mL/min) and risk factors such as SAH, DM, dyslipidemia, sedentary lifestyle, smoking, and a family history of CVD was also assessed. Data were collected using a standardized form developed for the Catarina Heart Study, to be completed by the attending physician. This form includes sociodemographic data, anthropometric measurements, dietary habits, clinical information, mental status assessment, procedures performed within the first 24 hours of admission, religiosity index, physical activity level, hemodynamic status, echocardiogram results, laboratory tests, and 1-year follow-up information.

Creatinine clearance was calculated using the Cockcroft formula,^17^ based on the first creatinine measurement obtained within 72 hours of hospital admission. Continuous creatinine clearance values were used to analyze both primary and secondary outcomes. For comparisons between renal function and cardiovascular risk factors, patients were categorized into two groups: those with renal dysfunction (GFR <60 mL/min) and those with preserved renal function (GFR ≥60 mL/min).^18^

A sample size of 458 patients was calculated to ensure 90% power and a 5% alpha to detect a 24% incidence of MACEs in patients with renal dysfunction, compared to 12% in those without, based on the data reported by Gallacher et al.^19^

The study was approved by an institutional ethics committee and complied with all ethical and legal requirements.

### Statistical analysis

Data were entered into Microsoft Excel spreadsheets and subsequently analyzed using the SPSS software version 13.0 (Chicago: SPSS Inc; 2005). Descriptive statistics were performed for all variables. Categorical data were presented as absolute and relative frequencies, while continuous data were expressed as measures of central tendency (mean or median) along with their respective measures of variability (standard deviation or interquartile range [IQR]). The Kolmogorov-Smirnov test was used to assess the normality of continuous variables. For bivariate analysis, associations between the dependent and independent variables were evaluated using the chi-square test. The association between renal function and clinical outcomes was analyzed using Cox regression, incorporating creatinine clearance as a continuous variable (calculated using the Cockcroft-Gault formula). The model also included DM, dyslipidemia, SAH, family history of CVD, smoking status, sedentary lifestyle, body mass index (BMI), and age. A p-value of <0.05 was considered statistically significant.

## Results

The study included 1,324 patients who experienced their first AMI between 2016 and 2023. The mean age was 60.9 ± 11.4 years, and 67.4% of participants were male. Among the cohort, 58.9% had SAH, 27.1% had DM, 32.7% had dyslipidemia, 32.3% were smokers, and 45.9% had a family history of CAD. The median creatinine clearance was 89.5 mL/min (IQR: 66.2-116.6). Table 1 shows additional patient characteristics.

**Table 1:** – Patients’ baseline characteristics

Characteristics	N (%)
LVEF, mean±SD	51.3±12.5
Age (years), mean±SD	60.9±11.4
Alcohol use	418 (31.6)
Diabetes mellitus	358 (27.1)
Dyslipidemia	431 (32.7)
Systemic arterial hypertension	778 (58.9)
Family history	606 (45.9)
Sedentary lifestyle	818 (61.9)
Male	893 (67.4)
Smoking	421 (32.3)
Creatinine clearance (mL/min), median (IQR)	89.5 (66.2-116.6)
LVEF, median (IQR)	53.00 (43.0-61.0)
BMI (kg/m2), median (IQR)	27.3 (24.5-30.5)
SYNTAX score, median (IQR)	13.00 (7.0-20.0)

BMI: body mass index; CAD: coronary artery disease; IQR: interquartile range; LVEF: left ventricular ejection fraction; SD: standard deviation.

Creatinine clearance <60 mL/min was associated with a higher prevalence of SAH and DM. Further details are provided in Table 2.

**Table 2:** – Association between creatinine clearance and risk factors

Risk factors	Clearance <60 mL/min, n (%)	Clearance >60 mL/min, n (%)	p-value
Alcohol use	38 (19.5)	373 (34.0)	<0.001
Diabetes mellitus	80 (40.8)	268 (24.5)	<0.001
Dyslipidemia	76 (38.8)	344 (31.4)	0.043
Systemic arterial hypertension	156 (79.6)	604 (55.1)	<0.001
Familial history	70 (35.7)	523 (47.7)	0.002
Smoking	45 (23.2)	364 (33.6)	0.004
Sedentary lifestyle	34 (28.3)	455 (38.7)	0.025
Male	143 (73.0)	732 (66.5)	0.077

In the analysis of 1-year outcomes, higher creatinine clearance was significantly associated with a lower hazard ratio (HR) for MACEs (HR: 0.992 per 1 mL/min increase; 95% CI: 0.984-0.999; p=0.030). Higher clearance was also linked to significantly reduced HR for all-cause mortality. Additional associations are presented in Table 3 and the Central Illustration.

**Table 3:** – Association between clearance values and 1-year outcomes

Outcomes	Hazard ratio (95% CI)	p-value
Unstable Angina	1.002 (0.993-1.011)	0.204
Stroke	0.991 (0.974-1.008)	0.293
AMI	0.991 (0.981-1.001)	0.072
MACE	0.992 (0.984-0.999)	0.030
Cardiovascular mortality	0.994 (0.981-1.008)	0.407
All-cause mortality	0.984 (0.970-0.998)	0.021
Stent restenosis	0.996 (0.979-1.014)	0.617
Readmission	0.996 (0.991-1.001)	0.127
Stent acute thrombosis	1.002 (0.990-1.014)	0.763

AMI: acute myocardial infarction; CI: confidence interval; MACE: major adverse cardiovascular events.

## Discussion

This study presents important findings on the incidence of mortality and cardiovascular outcomes within 1 year in Brazilian patients diagnosed with AMI, stratified by creatinine clearance at admission. The results demonstrate a significant association between impaired renal function and an increased HR for both all-cause mortality and MACEs.

The patient sample was predominantly male, with a mean age of approximately 61 years, and more than half of the individuals had SAH — findings consistent with other studies.^20-23^ Regarding creatinine clearance values, previous studies assessing renal function in patients post AMI reported lower medians than those observed in this study, ranging from 63.7 to 77 mL/min/1.73 m^2^.^22,24-26^ The association between DM, SAH, and renal dysfunction observed in this study was expected. DM is one of the main etiological factors for kidney damage, and this finding is consistent with previous reports.^27,28^ SAH can act as both a cause and a consequence of declining renal function, and both conditions are independent risk factors for CVD.^29^ The association between dyslipidemia and kidney disease may be explained by a potential link to increased atherosclerosis, including renal atherosclerosis. However, it is also possible that this association is spurious and the result of chance.^30,31^

The present study is one of the largest Brazilian investigations to demonstrate that renal function at admission is a prognostic factor in patients with AMI. Higher creatinine clearance values are associated with lower HRs for MACEs and all-cause mortality. This association was confirmed through multivariate analysis, which included variables such as DM, age, and BMI — potential confounders — and incorporated absolute clearance values. In other words, lower creatinine clearance in AMI patients is an independent predictor of worse outcomes, regardless of the presence of overt renal dysfunction.

Previous research already suggests that renal function at admission is associated with worse prognosis in patients with AMI. Evidence indicates that serum creatinine concentration at admission is a strong predictor of in-hospital mortality in patients with ST-segment elevation myocardial infarction, particularly among younger and female patients.^32^ Another study showed that an estimated GFR (eGFR) below 60 mL/min/1.73 m^2^ at admission is associated with increased early and late mortality in AMI patients who develop acute kidney injury (AKI).^33^ Furthermore, creatinine clearance calculated at admission has been identified as an independent predictor of long-term mortality in AMI patients, with an increased risk of death up to 10 years after the event.^34^ Reduced renal function at admission has also been linked to higher mortality rates in patients undergoing percutaneous coronary intervention.^35^

On the other hand, some studies, although showing an association between lower GFR and higher 30-day mortality after AMI, also found that percentage changes in clearance were not significantly associated.^26^ Additionally, other authors have explored more indirect links between renal dysfunction and MACE, which may be explained by comorbidities commonly found in patients with AKI that also contribute to worse outcomes, such as anemia.^36^

These data reinforce the importance of assessing renal function, which is often overlooked in the management of patients with AMI. The association between renal function at admission and clinical outcomes suggests that evaluating creatinine clearance could serve as an early prognostic tool. This would allow for the identification of high-risk patients for cardiovascular adverse events and mortality, enabling more targeted interventions that may improve long-term outcomes.

Several limitations of the present study should be acknowledged. First, it is based on exploratory analyses of an existing database, which may have inherent limitations regarding data completeness and consistency since the database was not designed specifically to address the research questions of this study. Despite the researchers receiving appropriate guidance and training, data collection may have been subject to measurement biases due to the challenges of conducting interviews in an emergency setting.

Although the sample size was calculated to be sufficiently large, a formal power analysis was not conducted, and the study may have lacked statistical power to detect smaller effect sizes for some secondary outcomes. Additionally, critically ill patients were not included due to their inability to participate in the interview process, which may have introduced selection bias and limited the generalizability of the results, particularly to the most severely ill patients with AMI.

Moreover, certain biomarkers associated with cardiovascular risk, such as albuminuria, uric acid, and phosphate levels, were not available in the dataset, limiting the ability to assess their contribution to post-AMI prognosis. There may also have been unmeasured confounding factors, such as medication adherence or socioeconomic status, that influenced the observed associations.

Finally, the possibility of a type 1 error cannot be ruled out, as the results may have been influenced by chance or unknown biases. Confounding by indication may also have occurred, as patients with worse renal function might have received different treatment strategies that independently affected the outcomes.

## Conclusion

The findings of this study indicate that higher creatinine clearance values at admission are directly associated with a lower risk of MACEs and all-cause mortality within 1 year following AMI. These results suggest that renal function, specifically assessed through creatinine clearance, may serve as a valuable prognostic marker for predicting long-term cardiovascular outcomes in patients with AMI. Given the simplicity and cost-effectiveness of creatinine clearance measurement, its integration into routine clinical practice could support the early identification of high-risk patients who may benefit from more intensive monitoring and therapeutic interventions.
